# Towards Understanding Aerogels’ Effect on Construction Materials: A Principal Component Analysis Approach

**DOI:** 10.3390/gels9120935

**Published:** 2023-11-28

**Authors:** Emil Obeid, Hamdi Chaouk, Rabih Mezher, Eddie Gazo Hanna, Omar Mouhtady, Jalal Halwani, Khaled Younes

**Affiliations:** 1 College of Engineering and Technology, American University of the Middle East, Egaila 54200, Kuwait; emil.obeid@aum.edu.kw (E.O.); hamdi-chaouk@aum.edu.kw (H.C.); rabih.mezher@aum.edu.kw (R.M.); eddie-hanna@aum.edu.kw (E.G.H.); omar.mouhtady@aum.edu.kw (O.M.); 2 Water and Environment Sciences Laboratory, Lebanese University, Tripoli P.O. Box 6573/14, Lebanon; jhalwani@ul.edu.lb

**Keywords:** construction materials, concrete, machine learning, PCA, materials science

## Abstract

This study investigates the applicability of Principal Component Analysis (PCA) for distinguishing construction materials. The approach enhances data presentation, revealing distinct clusters and variable impacts on materials. This perspective provides valuable insights into concrete materials, guiding materials science and engineering practices. Our findings show the capacity of PCA to show a clear distinction between concrete and non-concrete composites. Compressive strength significantly affects certain composites, being influenced by aerogel loading. The peculiar role of aerogel density among the other factors is attributed to their possession of the smallest thermal conductivity. To address moderate total variance of PCA, segregation into concrete (C) and non-concrete (NC) categories is explored, offering a more robust distinction and higher clustering. Concrete materials show higher variance, emphasizing the effectiveness of the segregation approach. PCA highlights aerogel density’s influence on thermal conductivity on concrete materials. For non-concrete materials, a moderately higher variance is noted, emphasizing the critical role of aerogel-related properties (size and density). These findings underscore the importance of aerogel characteristics in shaping material behaviour.

## 1. Introduction

The surging global population and growing human needs have led to a significant surge in energy consumption and demand. Consequently, there is a pressing need for more energy-efficient and energy-saving buildings. Previous research indicated that the building sector alone is poised to consume an estimated 35–40% of the total energy supply [1,2]. Furthermore, the building sector stands out as the largest contributor to greenhouse gas emissions, causing detrimental effects on the environment [3,4].

The energy consumption of a building is primarily influenced by its thermal insulation, which is crucial for achieving advanced energy efficiency. The demand for thermal insulating and sustainable cementitious composites is on the rise as a means to reduce energy consumption and enhance buildings’ thermal insulation. Various types of thermal insulating lightweight aggregates, such as expanded glass beads [5], expanded polystyrene [6], expanded perlite [7], and expanded clay [8], are incorporated into cementitious composites to improve thermal insulation properties. Among these, aerogels emerge as an exceptional choice due to its remarkable thermal insulating effect and lightweight characteristics [9], with its thermal conductivity even surpassing that of still air [10]. Numerous research studies have explored the incorporation of aerogel powder or particles into cementitious thermal insulating composites, consistently demonstrating such composites’ superior thermal insulation properties compared with traditional lightweight concrete without aerogels [9,11,12,13,14,15,16]. This innovation shows promise in addressing the challenge of lowering energy consumption and meeting thermal insulation requirements. According to the literature, employing aerogel-incorporated cementitious composites for exterior walls can result in energy savings of 5–6% for space heating and cooling [15]. The particle size of an aerogel is crucial in determining its density, thermal, and acoustic properties. Smaller aerogel granules have better density and improved thermal insulation qualities, with a significant 17% reduction in thermal conductivity when compared with larger granules [17]. While aerogels are frequently utilized and explored in thermal insulating composite materials due to its lightweight density and thermal insulation qualities, it is critical to recognize its inherent brittleness and lower strength properties. Many studies on aerogel-based cementitious composites have been carried out [18,19,20,21,22,23,24,25], with some researchers attempting to improve both their thermal insulation and mechanical qualities by including materials such as graphene [26], carbon nanotubes [27,28], and attapulgite nanofiber [29]. There are several forms of aerogels on the market, including cellulose aerogels [30], graphene aerogels [31], carbon aerogels [32], and silica aerogels [21,33], with silica aerogels being the most common and cost-effective alternative. Aerogels have much lower heat conductivity than traditional thermal insulation materials such as foam glass, cotton wools, polystyrene foam, mineral wools, and polyurethane foam [34]. However, the incorporation of hydrophobic silica aerogels in cementitious composites may lead to reduced strength [35] and flowability [16] due to t lower adhesive properties and fragile nature. The lightweight density and brittleness of aerogels present challenges in achieving a homogeneous mix between aerogel and cementitious composites.

Numerous efforts have been devoted to conducting a comprehensive comparison of construction materials, assessing their performance, technical attributes, cost-effectiveness, and future availability (sustainability). These comparative analyses align with an emerging concept known as “Materials Ecology” [36,37]. However, challenges may arise when comparing materials with closely proximate properties and dealing with the intercorrelation of various factors involved in the assessment [36]. Principal Component Analysis (PCA) finds its application in handling data with intercorrelated variables. Functioning as an unsupervised learning tool, PCA proves to be a robust technique for reducing dimensionality in a given dataset and mitigating intercorrelations among the considered factors by generating new orthogonal variables, termed principal components (PCs) [38]. This attribute is particularly noteworthy due to its substantial advantages in addressing the multicollinearity of variables at hand [38].

The objective of this work is to utilize Principal Component Analysis (PCA) to identify correlations between the overall characteristics of construction materials and the unique attributes of aerogels. If such connections are identified, it will enhance our capacity to interpret and comprehend the various factors that impact the suitability of a particular aerogel for use in construction materials. The insights derived from PCA can inform the application of aerogels in construction, encompassing aspects like manufacturing methods, experimental conditions, and the insulation effectiveness of the selected materials.

## 2. Results and Discussion

### 2.1. PCA Presentation of Concrete and Non-Concrete Aerogel Materials

**Figure 1:**
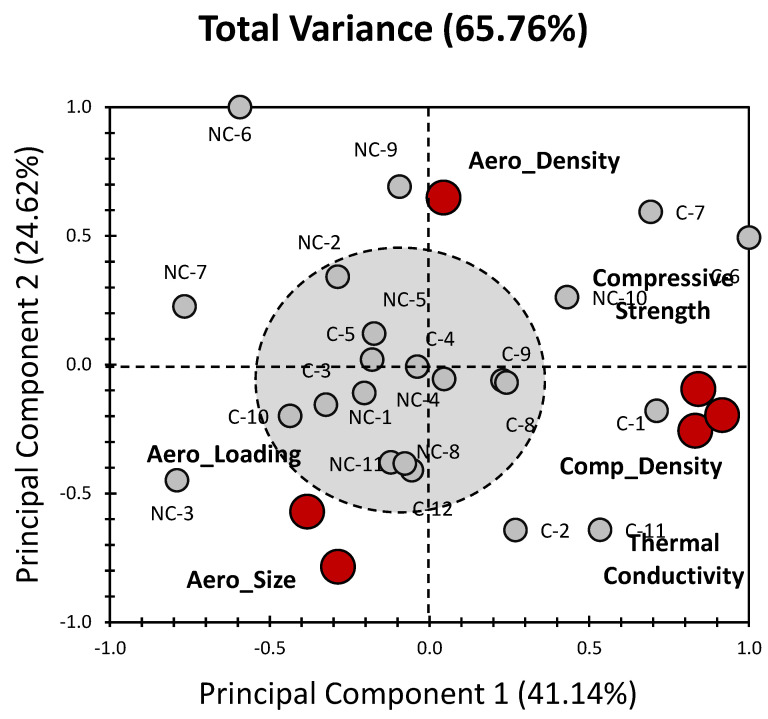
Presents PCA biplot representation of all datasets for the properties of aerogels incorporated in cementitious composites (data were obtained from the previous findings of Adhikary et al. [39]). Grey bullets indicate the different composites under investigation (C_x_: concrete composites, and NC_x_: non-concrete composites). Red bullets indicate physical properties of composites (aerogels’ specific properties and bulk properties).

Figure 1 illustrates the results of the PCA we carried out to investigate data previously reported by Adhikary et al. [39]. In this analysis, the studied population is categorized into two primary groups: concrete composites (C_x_) and non-concrete composites (NC_x_). The first two principal components (PCs) together presented a substantial portion of the total variance, amounting to 65.76% (with PC_1_ contributing 41.14% and PC_2_ contributing 24.62%, as displayed in Figure 1). Remarkably, the PCA highlights a clear distinction between the two groups, with concrete composites predominantly clustering towards the right side of the PCA plot (with the exception of C_3_, C_5_, C_10_, and C_12_). Conversely, non-concrete composites tended to aggregate towards the left side of the biplot (with the exception of NC_10_). This conspicuous separation, coupled with the moderate-to-high variance evident in the PCA analysis, underscores its efficacy in comparing different composite materials and, consequently, uncovering latent patterns related to their physical properties and behaviour. Significantly, the PCA analysis does not reveal any notable clustering among the various types of composites. Instead, a prominent and expansive cluster is discernible around a central node (as depicted in Figure 1). This suggests that the analysed composites exhibit a degree of heterogeneity, with no distinct subgroups or associations among them, underscoring the need for further investigation to fully comprehend the factors influencing their characteristics.

**Figure 2:**
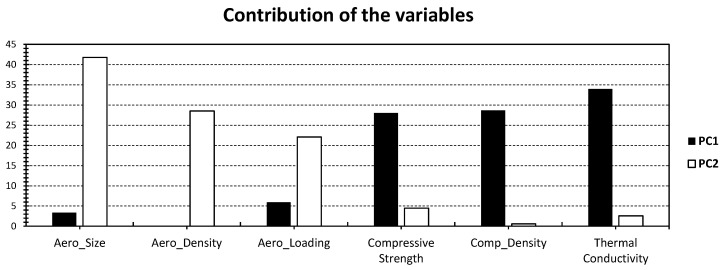
% Contribution of the different variables in Figure 1 relative to PC_1_ (black) and PC_2_ (white).

In the context of the physical properties analysed (variables of the PCA; Figure 2), it is intriguing to note that PC_1_ displayed the most substantial loadings for specific attributes, particularly compressive strength, composite density, and thermal conductivity (with approximately 28% for composite density and compressive strength and 34% for thermal conductivity, as shown in Figure 2). Furthermore, there was a minor contribution from the unique properties of the aerogel material. Conversely, PC_2_ showcased a different pattern with a more prominent emphasis on the aerogel’s distinctive characteristics. Notably, the most dominant factor was identified as the aerogel size, contributing significantly at 42% (Figure 2). This was due to the moderate-to-high contributions of aerogel density (around 28%) and aerogel loading (approximately 22%), as depicted in Figure 2.

This marked divergence in loadings between the Aerogels specific properties (including Aero_Size, Aero_Density, and Aero_Loading), on one hand, and the bulk properties of the material (namely compressive strength, composite density, and thermal conductivity) on the other hand suggests a clear distinction in their influence on the overall composition and behaviour of the composites. In fact, the “segregation” seen in Figure 1 between the specific and bulk properties is consistent with the literature. For example, it was reported that the compressive strength of a composite decreases with an increase in the aerogel loading in the cement [9,40]. Moreover, the aerogel density and the thermal conductivity follow opposite trends with respect to each other, where according to the works of Buratti et al. [17], a larger aerogel density leads to a reduction in the thermal conductivity and better thermal insulation. However, the larger the size of the aerogel, the higher the thermal conductivity [17]. From a first impression, it seems that this latter behaviour is not completely evidenced in Figure 1 (segregation between Aero_Size and thermal conductivity). Nonetheless, as the aerogel size expands, there is a slight increase in thermal conductivity, but it remains relatively low. This means that thermal insulation is somewhat reduced, though it still exists [17]. It is worth mentioning that an increase in the aerogel loading causes a decrease in the thermal conductivity of the composite [39].

The particle size of an aerogel is a crucial determinant of its physical characteristics, influencing its density, thermal, and acoustic attributes. Finer particles of aerogel exhibit increased density and enhanced thermal insulation capabilities, with studies indicating a 17% decrease in thermal conductivity for smaller granules compared with those of a larger size [17]. Tsioulou et al. [41] investigated the effect of an aerogel’s particle size on the thermal properties of aerogel-integrated cementitious materials. The research disclosed that cement mortar produced with aerogel beads at the same aerogel volume fraction exhibited a decreased thermal conductivity compared with the composite mortar produced with aerogel powder. At a 30% aerogel fraction, the thermal conductivity is recorded as ~1.15 W/(m·K) for mortar with aerogel beads and approximately 1.25 W/(m·K) for mortar with aerogel powder. This reversed proportionality is further validated, in our case, by the negative correlation between thermal conductivity and aerogels’ physical features (Figure 1). These findings highlight the multifaceted nature of the physical properties under investigation and the need to consider both bulk and specific attributes when evaluating and considering the characteristics of composite materials [39].

When we consider both the composite materials (individuals) and their corresponding physical properties (factors) together, as depicted in Figure 1, several patterns emerge. Notably, a substantial clustering effect becomes apparent, primarily involving bulk properties such as composite density, thermal conductivity, and compressive strength. These particular factors exhibit a robust positive influence, especially for C_1_, C_6_, and C_7_, as they align predominantly along PC_1_ (Figure 1). Concerning the compressive strength, and as previously mentioned, this property affects C_6_, and C_7_ more than it affects C_1_, as shown in Figure 1; indeed, the aerogel loading for C_6_, and C_7_ is 60% (Table 1), which is more than 40%. The latter magnitude of loading can reduce the compressive strength of cementitious composite beyond the threshold values suggested by the CEB requirements for structural concrete [13,40].

From a literature point of view, referencing the work by Gao et al. [9], an incremental rise in aerogel content from a null value to 60% was associated with diminished mechanical integrity of the composite material. The density of the composite decreased from a standard 1980 kg/m^3^ to 1000 kg/m^3^ when aerogel made up 60% of the volume, with a corresponding compressive strength reduction from roughly 75 MPa to 8.3 MPa. This represents a halving of density and a decrease in compression strength to one ninth of the initial value for concrete without aerogel content. These findings are consistent with reductions in compressive strength noted in various other studies [12,14,16,40,42,43,44,45,46,47]. In our case, it can be confirmed by the high disparity yielded by the general physical properties, on one hand, and aerogel features on the other.

On the other hand, when focusing on the aerogel’s specific properties, a distinct and highly correlated cluster comes into view for Aero_Size and Aero_Loading. This cluster exhibits a marked positive influence along the negative side of PC_2_, affecting mostly C_2_ and C_11_ from the concrete group and mostly NC_3_, NC_8_, NC_11_, and NC_12_ from the non-concrete group, as seen in Figure 1. Regarding Aero_Loading, its influence is expected for most cementitious composites since increases in aerogel concentration govern the reduction in thermal conductivity [9,40,48,49] and compressive strength [12,14,16] for these composites and seem to be more prominent for the latter elements of the concrete and non-concrete group.

Interestingly, Aero_Density stands out as an exception, as it is singularly positioned on the positive side of PC_2_, with a comparatively negligible contribution along PC_1_. Nonetheless, Aero_Density still shows a substantial influence, particularly on NC_9_ (Figure 1). The impact that Aero_Density has on NC_9_ could be attributed to the fact that high-density aerogel granules have been associated with better thermal insulation [17]. Even though the density of NC_9_ is not available in Table 1, is thermal conductivity of 0.265 W/m·K is among the lowest for the non-concrete composites listed in Table 1.

This comprehensive analysis underscores the intricate interplay between the composite materials and their specific physical properties, illuminating the distinct clusters and influential factors that shape their overall behaviour and characteristics [39]. In brief, our analysis did not reveal any significant differentiation among the investigated composite materials. However, a relevant distinction emerged between concrete and non-concrete materials along the principal component axis PC_1_. It is more likely that the variations observed in PC_1_ are primarily driven by differences in bulk properties. This observation aligns with the known distinctions in physical characteristics between these two material categories [39]. When we delve into the individual variables, a striking discrepancy becomes apparent between the bulk properties and the unique characteristics of aerogels. Consequently, this separation along the first two PCs suggests their independence, a notion further underscored by the orthogonality between the principal components [38]. The variability exhibited in our findings is substantiated by the moderate-to-high variance we observed in the data. However, the limited clustering effect raises concerns regarding our ability to effectively distinguish between the various investigated materials. To address this issue, we may consider exploring alternative strategies for segregating individuals based on whether they belong to the concrete (C) or non-concrete (NC) categories. This could provide a more robust means of categorization and enhance our ability to discern meaningful differences within the investigated construction materials. Further analysis is warranted to refine our understanding of these material properties and their implications.

### 2.2. PCA Presentation of Concrete Aerogel Materials

**Figure 3:**
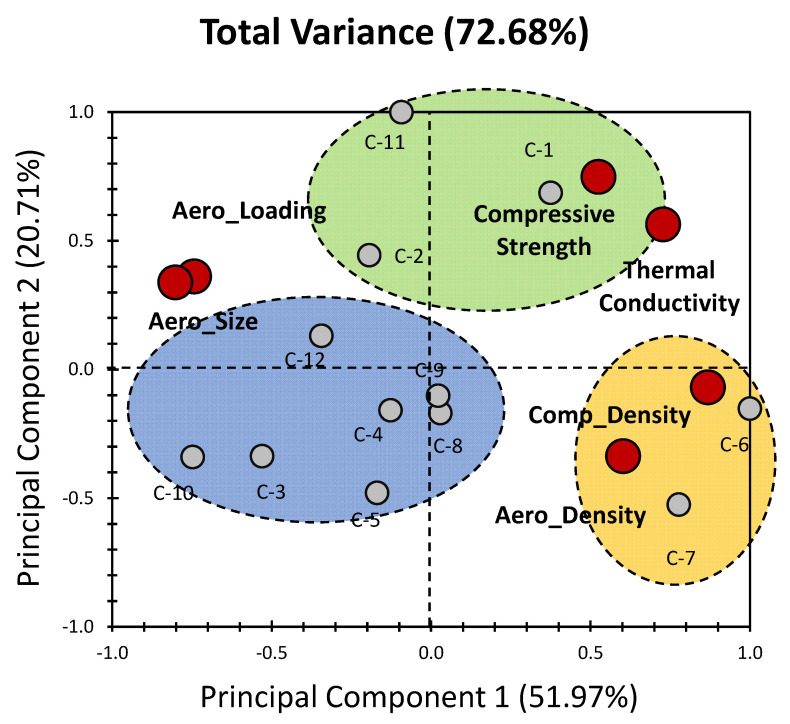
PCA biplot representation of concrete materials for the properties of aerogels incorporated in cementitious composites (data were obtained from the previous findings of Adhikary et al. [39]). Grey bullets indicate the different composites under investigation (C_x_: concrete composites). Red bullets indicate physical properties of composites (aerogels’ specific properties and bulk properties).

**Figure 4:**
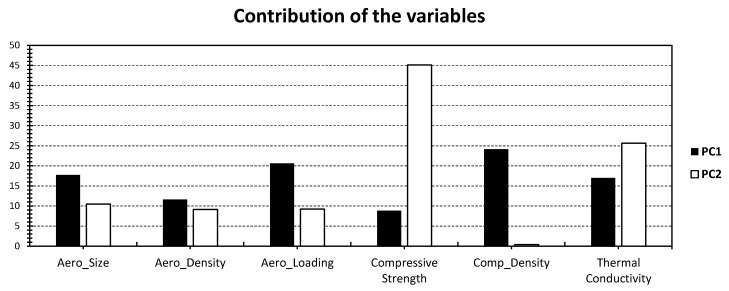
Contribution of the different variables in Figure 3 relative to PC_1_ (black) and PC_2_ (white).

Figure 3 provides an insightful visual representation of the PCA results, specifically focusing on the concrete materials (C_x_) within the dataset as originally reported by Adhikary et al. [39]. One striking observation from this analysis is the higher variance compared with the all-in-one dataset approach. In fact, the first two PCs collectively account for a substantial 72.68% of the variance, with PC_1_ contributing 51.97% and PC_2_ contributing 20.71% (as depicted in Figure 3). This significant variance underscores the effectiveness of segregating the individuals into two distinct groups. Notably, this approach results in a more discernible distribution of data points compared with the initial unified dataset. Three distinct clusters emerge in Figure 3 (blue, orange, and green), further validating the efficiency of this separation technique. The clusters suggest that the concrete materials within the dataset exhibit notable distinctions, likely stemming from underlying variations in their characteristics. For variables, their contributions to the PCs reveal some intriguing insights. It is evident that PC_1_ has a considerable influence, with variables contributing between 9% and 24% (as illustrated in Figure 4). This suggests that all the investigated variables exert a relatively similar magnitude of impact on the concrete materials under investigation. Conversely, PC_2_ exhibits a different pattern, with compressive strength emerging as the most dominant factor, contributing a substantial 45% to this component. Thermal conductivity also plays a moderate role, contributing 25% to PC_2_ (Figure 4). These variable contributions provide valuable information about the underlying factors that contribute to the variability within the concrete materials.

Within the realm of concrete materials, our PCA analysis revealed the emergence of three distinct clusters, as depicted in Figure 2 Each of these clusters sheds light on unique patterns and influences within the dataset. The blue cluster, encompassing the materials C_3_, C_4_, C_5_, C_8_, C_9_, C_10_, and C_12_, portrays an intriguing pattern. These materials exhibit a negative influence along PC_1_, suggesting they are characterized by properties that tend to decrease as we move along this PC. It is noteworthy that the individuals within this cluster are positively influenced by Aero_Loading and Aero_Size along the negative side of PC_1_. This implies that the variations in these two variables contribute to the separation of this cluster along the primary axis of variation. In Table 1, it can be noticed that C_3_ and C_12_ possess the highest aerogel loading (40%) among the individuals of these clusters, which is logical since these materials are approximately the closest to Aero_Loading. And it can be seen from Figure 3 that, on average, the further these materials are from Aero_Loading, the lower the aerogel concentration is (Table 1). Furthermore, it can be seen that the property that decreases as we move in this cluster along the negative PC_1_ is the thermal conductivity, with its values decreasing (Table 1) from approximately 1.135 W/m·K (C_9_) to 0.33 W/m·K (C_10_). This implies that the variations in these two variables (Aero_Loading and Aero_Size) contribute to the separation of this cluster along the primary axis of variation. Along PC_2_, Aero_Density and Comp_Density exert a positive influence on these materials. Again, this could be attributed to the influence that Aero_Density has on the decrease in the thermal conductivity [17]; in Table 1, we can see the two lowest values for C_3_ and C_10_, 0.32 W/m·K and 0.33 W/m·K, respectively, while C_3_ and C_10_ are approximately at the same y-coordinate of Aero_Density. Meanwhile, higher values for the thermal conductivity are reported for C_4_, C_5_, C_8_, C_9_, and C_12_, which are at an offset (Figure 3) with respect to Aero_Density. These findings highlight the complex interplay of factors affecting the concrete materials in the blue cluster (Figure 3).

Conversely, the yellow cluster, consisting of the materials C_6_ and C_7_, exhibits a different trend. These materials showcase a positive influence along PC_1_ indicative of characteristics that tend to increase along this component. Simultaneously, they display a low to moderately negative influence along PC_2_. Within this cluster, both Aero_Density and Comp_Density positively influence the materials. This is notably present for C_6_ and C_7_, where it could be reasoned that along PC_1_ or PC_2_ (since Aero_Density contributes to 11% and 9% of the variables along PC_1_ and PC_2_, respectively, which are comparable percentages), they are the closest to Aero_Density with the highest aerogel density at 130 kg/m^3^. However, Aero_Loading and Aero_Size present a negative influence on C_6_ and C_7_ along PC_1_ and PC_2_. However, Aero_Loading and Aero_Size, present a negative influence on C_6_ and C_7_ along PC_1_ and PC_2_. This suggests that the variables associated with aerogel and composite density play a significant role in distinguishing the yellow cluster (Figure 3).

The green cluster, which includes the materials C_1_, C_2_, and C_11_, provides yet another facet of the dataset. In this case, the influence is positive along PC_2_, with a minor negative-to-moderate positive influence along PC_1_. This implies that the primary variation within this cluster occurs along the second principal component, distinguishing these materials based on factors related to PC_2_. The closest materials to the compressive strength bullet are C_1_, and C_11_ which makes sense since they have the highest compressive strengths, at 77.5 and 75 MPa, respectively (Table 1). Also, it can be concluded from these findings that C_1_ and C_11_ are the least suitable candidates among the herein-studied concrete aerogel materials for use in thermal insulating purposes, since they have the highest mechanical properties as well as some of the highest thermal conductivities (1.35 W/m·K for C_1_ and 1.26 W/m·K for C_11_). And according to the literature [39], most aerogels are highly effective thermal insulating materials with low mechanical properties, and they are commonly used for thermal insulating applications. The interplay of variables that shape this cluster’s characteristics is distinct from that influencing the other clusters, suggesting that the composition and properties of materials within this group are notably different (Figure 3).

In summary, the segregation of concrete materials into these three clusters offers valuable insights into the underlying factors that contribute to their distinct characteristics. The varying influences of aerogel loading, size, and density, as well as composite density, underscore the complexity of material interactions within these clusters. These findings have the potential to inform aerogel-based concrete development efforts, with practical implications for material engineering and design. Further investigation of these clusters and their unique properties can yield deeper insights and guide more targeted approaches for enhancing concrete materials [39].

### 2.3. PCA Presentation of Non-Concrete Aerogel Materials

**Figure 5:**
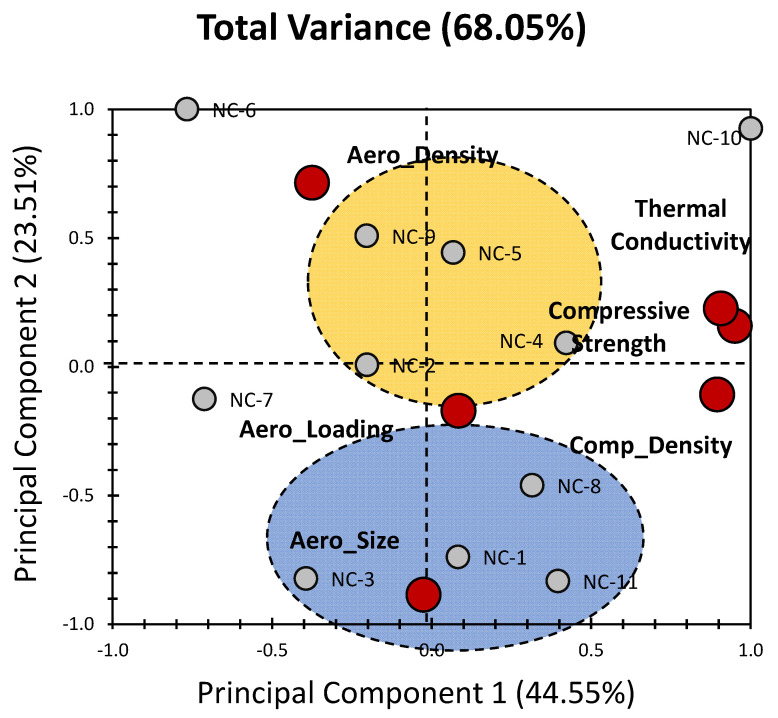
PCA biplot representation of non-concrete materials for the properties of aerogels incorporated in cementitious composites (data were obtained from the previous findings of Adhikary et al. [39]). Grey bullets indicate the different composites under investigation (NC_x_: concrete composites). Red bullets indicate physical properties of composites (aerogels’ specific properties and bulk properties).

**Figure 6:**
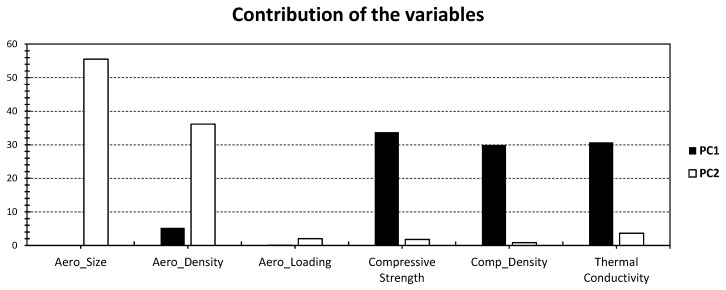
% Contribution of the different variables in Figure 5 relative to PC_1_ (black) and PC_2_ (white).

Figure 5 provides an illustration of the PCA results, with a specific focus on non-concrete materials (NC_x_), expanding upon the earlier findings of Adhikary et al. [39]. The first two PCs collectively capture a moderate-to-significant portion of the total variance, explaining 68.05% of the variance (with PC_1_ contributing 44.55% and PC_2_ contributing 23.51%; Figure 5). While this explained variance may appear slightly lower when compared with the PCA results for concrete aerogel materials (as seen in Figure 3), it moderately surpasses the variance explained by the all-inclusive PCA approach (as depicted in Figure 1). This suggests that the non-concrete materials exhibit a more nuanced and complex relationship with the PCs, resulting in a more dispersed distribution. This could be reflected by the lower level of clustering observed. Hence, unlike the broader spectrum of clusters evident in Figure 1, only two distinct clusters can be seen in Figure 5. This reduction in the number of clusters can be attributed to the substantial dissimilarity between aerogel-related properties and the bulk material properties. This distinction in material characteristics can be explained by the unique and moderate contribution of specific properties to PC_1_, primarily compressive strength, composite density, and thermal conductivity, which account for approximately 30% each (Figure 6). On the other hand, PC_2_ is predominantly influenced by Aero_Size, accounting for 55% of the variance, and it also has a moderate contribution from Aero_Density, which accounts for 36% of the variance (Figure 6).

When examining the non-concrete materials (individuals; Figure 5), two main clusters are present, each characterized by a distinct set of properties and influences (Figure 5). The blue cluster encompasses the materials NC_1_, NC_3_, NC_8_, and NC_11_ and is strongly influenced by Aero_Size, with a lesser impact of Aero_loadings (Figure 5). These materials predominantly exhibit a strong influence along the negative side of PC_2_ and a moderately negative-to-positive influence along PC_1_. In contrast, the yellow cluster, which comprises the materials NC_2_, NC_4_, NC_5_, and NC_9_, displays a different pattern. These materials are characterized by a moderate-to-low influence along the positive side of PC_2_ and a moderately negative-to-positive influence along PC_1_. Its localization between Aero_Density and Aero_Loading is particularly intriguing, as it suggests that these two properties play a pivotal role in shaping the characteristics of the materials within this cluster. It is worth emphasizing that the emergence of these two clusters underscores the substantial dependence of non-concrete materials on aerogel properties. This finding highlights the critical role that aerogel-related properties, such as Aero_Size and Aero_Density, play in determining the characteristics and behaviour of these materials [39]. The unique interplay between these properties and the principal components underscores their significance in shaping the overall performance and attributes of non-concrete materials.

In brief, the extended analysis of the PCA results presented in Figure 3 offers a deeper understanding of the relationships between non-concrete materials and their key properties. The identified clusters and their associated property influences shed light on the nuanced factors that drive the behaviour and characteristics of these materials. This knowledge could be instrumental in tailoring the design and development of non-concrete materials, optimizing their performance and enhancing their suitability for specific applications [39].

## 3. Conclusions

This study aims to estimate the applicability of the so-called “Principal Component Analysis” (PCA) for the distinction between materials involved in construction applications. In light of the findings, it becomes evident that the approach of separating individuals within the dataset enhances the efficacy of data presentation. The major findings of this study are presented below:

○ PCA revealed distinct clusters and varying impacts of different variables on the materials studied.
○ PCA provides valuable insights into concrete materials, their characteristics, and the factors influencing their differences, with practical implications for materials science and engineering.
○ PCA analysis of the entire dataset shows a clear distinction between two groups, with concrete composites predominantly clustering on the right side of the plot.
○ Compressive strength significantly affects C_6_ and C_7_, with aerogel loading at 60% impacting these composites more than C_1_, potentially reducing compressive strength below threshold values.
○ The peculiar role of aerogel density, aside from other factors, is attributed to its smallest thermal conductivity.
○ To overcome moderate variance in the “All Dataset” approach, an alternative strategy of segregating individuals into concrete (C) or non-concrete (NC) categories is considered.
○ Higher variance is noted when only concrete materials are considered (nearly 73%), emphasizing the effectiveness of segregating individuals into distinct groups.
○ PCA of concrete materials highlights the influence of aerogel density on the decrease in thermal conductivity (blue cluster).
○ Exclusive consideration of non-concrete materials yields a moderately higher variance (nearly 68%), emphasizing the critical role of aerogel-related properties (aerogel size and density) in determining characteristics and behaviour.

## 4. Materials and Methods

### 4.1. Data Collection and Normalisation

Data were collected from the published study of Adhikary et al. [39]. Table 1 presents an inventory of the different investigated materials, along with their compressive strength, composite density, and thermal conductivity and aerogel size, density, and loading.

**Table 1:** Properties of aerogel-incorporated cementitious composites.

Designated Composite	Compositions			Aerogel Size (µm)	Aerogel Density (kg/m^3^)	Aerogel Loading	Compressive Strength (MPa)	Density of Composite (kg/m^3^)	Thermal Conductivity W/(m⋅K)	Ref.
Concrete	Cement, GGBS, sand, aerogel	C-1	1		110	15	77.5		1.35	[41]
	Cement, silica fume, sand, aerogel	C-2	2	3000	100	30	35.15	1600	1.105	[9]
	Cement, silica fume, sand, aerogel	C-3	3	2250	125	40	18.5	1161	0.32	[12]
	Cement, sand, aerogel	C-4	4	1200	105	30		1500	0.505	[50]
	Cement, fly ash aggregate, sand, aerogel	C-5	5	2350	135	25.86	19.5		0.445	[18]
	Cement, sand, nano silica, aerogel	C-6	6	11	130	60%	48.725	1968.5	1.555	[13]
	Cement, sand, Nano silica, Silica fume, aerogel	C-7	7	11	130	60%	45.965	1910	0.885	[48]
	Cement, nongraded expanded perlite silica fume, sand, aerogel	C-8	8		110		18.6	1625	1.01	[48]
	Cement, graded expanded perlite silica fume, sand, aerogel	C-9	9		110		18.25	1535	1.135	[48]
	Cement, expanded glass, fly ash, aerogel	C-10	10	1500	70	21	7.96	787.95	0.33	[16]
High-performance concrete	Cement, sand, Silica fume, quartz, aerogel	C-11	11	2000	100	40	75	1300	1.26	[51]
	Cement, silica fume, aerogel	C-12	12	2000	100	40	35		0.6	[51]
Thermal insulating composite	Cement, fly ash, silica fume, aerogel	NC-1	13	2500	95	2.5	21.085	1050.5	0.335	[49]
GB-NSA cement-based composites	Cement, glass breads, nano silica aerogel	NC-2	14			3.75	15.5	928	0.184	[42]
Aerogel–cement-based coating	Cement, fly ash, silica fume, aerogel	NC-3	15	2000	100	65	3.5	658	0.17	[52]
Aerogel/cement composite	Cement, nano silica, aerogel	NC-4	16	15	80	33	39.35	1080	0.36	[43]
Cement–aerogel paste	Cement, fly ash, aerogel	NC-5	17		150	50	26.5		0.435	[43]
Foamed concrete	Cement, aerogel, foam	NC-6	18	250	170	23.9	2.5	449	0.115	[44]
Foamed concrete	Cement, GGBS, foam, nano aerogel	NC-7	19				0.78	310	0.09	[45]
Mortar	Cement, silica fume, aerogel	NC-8	20	2000	100	40	27.65		0.575	[11]
	Cement, aerogel	NC-9	21	6.5		1.25	9.5		0.265	[53]
	Cement, silica fume sand, aerogel	NC-10	22	2.6	125	32	46.65	1512.5	0.935	[54]
	Cement, silica fume sand, aerogel	NC-11	23	2500	100	30	22.575	1622.75	0.375	[55]

Each investigated variable carries a distinct weight in the data. To mitigate bias arising from magnitude differences, we employed a normalization technique akin to that utilized by Nimer et al. [56].

(1) $$X_{st}=\frac{\left(Value-Mean\right)}{Standard\;Deviation}$$

where “*X_st_*” presents the standardized dataset values.

### 4.2. Principal Component Analysis (PCA)

Following normalization, PCA results were obtained using XLSTAT 2014 software, employing a method consistent with the approach outlined by Younes et al. [46]. To address missing data in this study, a built-in feature was utilized, replacing absent values with the “Mode” based on the respective variables.

This study’s objective is to employ PCA on data from a prior study by Adhikary et al. [39] (Table 1). With PCA, we aim to uncover hidden patterns between the bulk properties of the investigated material and the specific properties of aerogels. This exploration enhances the interpretation and understanding of factors influencing the applicability of aerogels in construction materials. The PCA output provides insights into implications for aerogels in construction, spanning manufacturing approaches, experimental conditions, and the isolation efficiency of the chosen materials. The application of PCA involves six different factors influencing the 23 investigated materials (Table 1). As a data-driven, unsupervised machine learning technique, PCA reduces the dataset for better visualization, revealing the hidden patterns through correlations (negative or positive), and the representativity of principal components (PCs) for the population at hand. The *j*th PC matrix (*Fi*) is expressed using a unit-weighting vector (*Uj*) and the original data matrix *M* with *m* × *n* dimensions (m: number of variables, *n*: number of datasets), as outlined [46]. The mathematical approach of PCA is as follows:

(2) $$Fi=U_{j}^{T}M=\sum_{i=0}U_{ji}M_{i}$$

where *U* is the loading coefficient and *M* is the data vector of size *n*. The variance matrix *M*(*Var*(*M*)) is obtained by projecting *M* to *U*, and it should be maximized, as follows:

(3) $$Var(M)=\frac{1}{n}\left(UM\right){\left(UM\right)}^{T}=\frac{1}{n}{UMM}^{T}U$$

(4) $$MaxVar(M)=Max\left(\left(\frac{1}{n}\right)\;{UMM}^{T}U\right)$$

Since $\frac{1}{n}{MM}^{T}$ is the same as the covariance matrix of *M*(*cov*(*M*)), *Var*(*M*) can be expressed as follows:

(5) $$Var\;\left(M\right)=U^{T}cov\;\left(M\right)\;U$$

The Lagrangian function can be defined, by performing the Lagrange multiplier method, as follows:

(6) $$L=U^{T}$$

(7) $$L=U^{T}cov(M)U-\delta (U^{T}U-I)$$

For (7), “*U^T^U − I*” is considered to be equal to zero, since the weighting vector is a unit vector. Hence, the maximum value of *Var*(*M*) can be calculated by equating the derivative of the Lagrangian function (*L*), with respect to *U*, as follows:

(8) $$\frac{dL}{dU}=0$$

(9) $$cov(M)U-\delta U=\left(cov(M)-\delta I\right)U=0$$

where *δ*: eigenvalue of *cov*(*M*); *U*: eigenvector of *cov*(*M*).

## Data Availability

Data is contained within the article.
